# Neuromodulation of lower limb motor pathways with trans-spinal direct current stimulation: an overview of current findings

**DOI:** 10.1080/07853890.2021.1896902

**Published:** 2021-09-28

**Authors:** Mariana Pereira, Sofia Rita Fernandes, Pedro Cavaleiro Miranda, Mamede de Carvalho

**Affiliations:** a Instituto de Fisiologia, Instituto de Medicina Molecular, Faculdade de Medicina, Universidade de Lisboa, Portugal; b Instituto de Biofísica e Engenharia Biomédica, Faculdade de Ciências, Universidade de Lisboa, Portugal

## Abstract

**Introduction:**

The spinal cord (SC) is a complex structure containing several neuronal circuits related with motor function of the upper and lower limbs, operating under the influence of higher centres. Central nervous diseases can change the responses of the spinal motor circuits leading to its dysfunction. Over the last decade, there has been a growing interest in the study of trans-spinal direct current stimulation (tsDCS) as a potential therapeutic tool to modulate spinal circuits through the application of electric currents delivered non-invasively [1]. Computational modelling studies are potentially useful to optimise electrodes montage in order to target current delivery to specific spinal region and pathways [2]. The aim of this study is to describe the more effective tsDCS electrode montages to maximise the modulation effects in lower limbs, as derived from a narrative review of the literature.

**Materials and methods:**

A literature narrative review was carried out through Pubmed database and manual search, considering the following selection criteria: research papers published in journals with impact factor in the areas of neuroscience, neurophysiology and biomedical engineering from 2008 to 2018; use of keywords related with the topic (tsDCS, trans- spinal, lumbar, thoracic, spinal cord, motor pathways, computational modelling); written in English. A qualitative analysis was performed over the literature selected considering the following items: electrode montage; tsDCS protocol; methods for assessing motor responses; observed changes; computational results; induced electric field (EF) distribution.

**Results:**

Published studies showed different neuromodulation effects on the motor responses of the lower limb. Whereas some studies reported a reduction of spinal motoneurons excitability using anodal tsDCS, others described higher motor unit recruitment with cathodal stimulation (T10–T12), suggesting modulation of Ia-motoneuron (MN) synapse. Other authors failed to replicate a modulatory effect applying tsDCS [3]. Modelling studies predicted EF magnitudes sufficient for neuromodulation in the montages previously applied in the spinal segments to be modulated when these are located between the electrodes [2]. **Discussion and conclusions:** Literature review indicates that experimental findings mainly depend on electrode polarity and position over the SC. tsDCS is a tool amenable to modulate motor circuits excitability in the spinal cord. This intervention can have clinical implications in the treatment of conditions like spasticity. However, future studies should consider EF magnitude and its orientation relative to spinal neurons. We propose that future clinical protocols should be guided by computational modelling to increase the chances of consistent positive results [4].
